# *Rhodotorula glutinis*—potential source of lipids, carotenoids, and enzymes for use in industries

**DOI:** 10.1007/s00253-016-7611-8

**Published:** 2016-05-21

**Authors:** Anna M. Kot, Stanisław Błażejak, Agnieszka Kurcz, Iwona Gientka, Marek Kieliszek

**Affiliations:** Department of Biotechnology, Microbiology and Food Evaluation, Faculty of Food Sciences, Warsaw University of Life Sciences, Nowoursynowska 159C, 02-776 Warsaw, Poland

**Keywords:** Oleaginous yeast, β-Carotene, Torulene, Torularhodin, Phenylalanine ammonia-lyase

## Abstract

*Rhodotorula glutinis* is capable of synthesizing numerous valuable compounds with a wide industrial usage. Biomass of this yeast constitutes sources of microbiological oils, and the whole pool of fatty acids is dominated by oleic, linoleic, and palmitic acid. Due to its composition, the lipids may be useful as a source for the production of the so-called third-generation biodiesel. These yeasts are also capable of synthesizing carotenoids such as β-carotene, torulene, and torularhodin. Due to their health-promoting characteristics, carotenoids are commonly used in the cosmetic, pharmaceutical, and food industries. They are also used as additives in fodders for livestock, fish, and crustaceans. A significant characteristic of *R. glutinis* is its capability to produce numerous enzymes, in particular, phenylalanine ammonia lyase (PAL). This enzyme is used in the food industry in the production of l-phenylalanine that constitutes the substrate for the synthesis of aspartame—a sweetener commonly used in the food industry.

## Introduction

Until recently, the yeasts of the genus *Rhodotorula* were primarily considered to be saprophytes that spoil food. In recent times, a large number of studies have been published on the biotechnological uses of these yeasts, which suggest that they may constitute important group of microorganisms that might be of importance in industries in the future. *Rhodotorula glutinis* is considered to be the typical species of this genus. These yeasts are capable of synthesizing numerous metabolites useful in industries, such as lipids, carotenoids, and enzymes. Their clear advantage is their capacity to grow and synthesize metabolites on substrates containing different industrial waste raw materials, which considerably elevates the economic profitability of biotechnological processes. This study presents a literature review on the possibility to obtain microbiological lipids, carotenoids, and enzymes from *R. glutinis* biomass and their potential use in industries. Moreover, the pathways of lipids and carotenoid biosynthesis and the influence of selected environmental factors on the efficiency of these processes are described.

## History, taxonomy, morphology, and physiology of *R. glutinis*

Over the years, the asexually reproducing, colored yeasts have been assigned to numerous genera, such as *Torula*, *Mycotorula*, *Torulopsis*, *Cryptococcus*, and even *Saccharomyces*. Francis Charles Harrison, a Canadian microbiologist, who worked on yeasts found in regional cheeses in the 1930s, was the first one to use the name *Rhodotorula* (Barnett 2004). The genus name originates from the word *rhodos* (red in Greek) and *torula* (feminine diminutive form of the Neo-Latin *torus*—bulge) (Krzyściak et al. 2007).

*Rhodotorula glutinis* is considered to be a typical species of this genus, and it was described by Georg Fresenius in 1850. The yeasts were isolated from the cream of sour milk and named *Cryptococcus glutinis* at that time (Barnett 2004). *R. glutinis* is included in the order Sporidiobolales, class Microbotryomycetes, and phylum Basidiomycota in the Fungi kingdom. Integrated Taxonomic Information System provides three synonyms of the Latin name of *Rhodotorula glutinis*: *R. rufula*, *R. glutinis* var. *rubescens*, and *R. gracilis* (ITIS Standard 2016). The following varieties are distinguished within the species: *Rhodotorula glutinis* (Fresenius) var. *glutinis*, *Rhodotorula glutinis* var. *dairenensis* (yeast isolated in 1922 by Saito from air) (Fell and Statzell-Tallman 1998), and *Rhodotorula glutinis* var. *salinaria* (yeast isolated in 1969 from salt) (Hirosawa and Takada 1969).

The majority of the yeasts included in the species are mesophilic, although some of them thrive under lower temperatures, and aerobic organisms. The cells are spherical, ellipsoidal, or elongated in shape. *R. glutinis* reproduce asexually by multilateral or polar budding; certain strains form residual pseudomycelium (Fell and Statzell-Tallman 1998).

These yeasts are capable of using many compounds as sources of carbon. They include glucose, galactose, sucrose, maltose, trehalose, ethanol, glycerol, and hexadecane. A characteristic feature of this genus is its lack of capacity to perform sugar fermentation. The cells produce urease and Q-10 coenzyme. They can grow in the presence of 10 % NaCl, but they do not tolerate glucose concentration above 50 % (Fell and Statzell-Tallman 1998).

*R. glutinis* colonies that grow on permanent malt medium exhibit characteristic coloration that depends on the type of strain and growth conditions. They can be of creamy, yellow, salmon, pink, orange, coral, and blood red in color. In liquid media, they grow in the form of orange ring or sediment (Fell and Statzell-Tallman 1998; Hernández-Almanza et al. 2014). The colored pigmentation of the cells is due to the production of large amounts of carotenoids, which are responsible for protecting the cells against the effect of singlet oxygen and excessive radiation of visible and UV light spectrum (Hernández-Almanza et al. 2014).

*Rhodotorula* yeasts occur in common in the environment. They are isolated from air, soil, grass, lakes, oceans, food (i.e., milk, fruit juices), human skin, and feces (Wirth and Goldani 2012). The majority of the representatives of the genus do not exhibit pathogenic properties, although opportunistic pathogens are found among them, which cause dermatophytoses, and are referred to as rhodotorulosis. The most common etiological factors of these infections are the strains of the species *Rhodotorula mucilaginosa* (Biswas et al. 2001).

## Lipid biosynthesis by *Rhodotorula glutinis*

In recent years, there has been an increased interest in developing new methods to obtain lipids from these yeasts. One such method includes the production of microbiological lipids, referred to as SCO in the literature (*single cell oil*) (Beopoulos and Nicaud 2012). In comparison to the production of vegetable and animal fats, this method is independent of climate, season, and geographical position of a country. Production cycle is short, thanks to the rapid growth rate exhibited by the microorganisms (Santos et al. 2013). Microbiological lipids can be used as food additives, diet supplements, and substitutions for precious fats. Microbiological oils can also be used as substrates in the so-called third-generation biodiesel production (Li et al. 2008; Papanikolaou and Aggelis 2011b; Papanikolaou et al. 2001, 2003; Ratledge and Cohen 2008).

The yeast *Rhodotorula glutinis* belongs to the group of oleaginous microorganisms (Table 1), which are defined as those that are capable of producing and accumulating over 20 % of lipids in dry cellular substance (Ratledge and Cohen 2008). Fat is stored in the lipid bodies (Ham and Rhee 1998), whose structure is similar in all oleaginous yeasts. The core consists of backup hydrophobic compounds (such as triacylglycerols, free fatty acids, and sterols), and it is surrounded by a layer of phospholipids bound to proteins (Fickers et al. 2005). In these yeasts, the lipid bodies consist of neutral lipids in the form of triglycerides, and the composition of phospholipids differs from this composition in other cellular organelles. This stems from the fact that they primarily consist of phosphatidylcholine (38.6 %) and phosphatidylserine (43 %) (Ham and Rhee 1998).

**Table 1:** Lipid content in the cell biomass of different yeast strains of *Rhodotorula glutinis*

Strain	Cultivation method	Carbon source	Nitrogen source	Lipid content (%)	References
L/24-2-1	Single-stage continuous	Molasses	Ammonium sulfate	39.0	Alvarez et al. (1992)
T216	Batch	Glucose	Yeast extract	60.9	Dai et al. (2007)
ATCC 204091	Batch	Glycerol	Ammonium chloride, yeast extract	25.0	Easterling et al. (2009)
ATCC 204091	Batch	Distillery wastewaters from the Tequila production process		27.0	Gonzalez-Garcia et al. (2013)
IIP-30	Fed-batch	Glucose	Ammonium sulfate, yeast extract	66.0	Johnson et al. (1992)
IIP-30	Fed-batch	Sucrose	Ammonium sulfate, yeast extract	50.0	Johnson et al. (1995)
NRRL Y-1091	Fed-batch	Glucose	Ammonium sulfate	40.0	Lee and Yoon (1990)
GM4	Batch	Glucose	Ammonium sulfate, yeast extract, ammonium tartrate	39.3	Li et al. (2013)
ATCC 204091	Batch	Nonhydrolyzed levoglucosan	Yeast extract	42.2	Lian et al. (2013)
CGMCC 2703	Fed-batch	Corncob hydrolysate	Ammonium sulfate	47.2	Liu et al. (2015)
CCMI 145	Batch	Glucose	Ammonium chloride, yeast extract	17.8	Lopes da Silva et al. (2011)
TISTR 5159	Batch	Glycerin fraction after diesel production		36.9	Louhasakul and Cheirsilp (2013)
NCYC 154G	Fed-batch	Glucose	Yeast extract	35.0	Ratledge and Hall (1979)
TISTR 5159	Fed-batch	Glycerol	Ammonium sulfate	60.7	Saenge et al. (2011b)
AY 91015	Batch	Glucose	Yeast extract	18.2	Wang et al. (2009)
CGMCC 2258	Fed-batch	Glucose	Monosodium glutamate wastewater	45.0	Xue et al. (2008)
CGMCC 2258	Fed-batch	Corn starch wastewater		35.0	Xue et al. (2010)
BCRC 22360	Batch	Glycerol	Thin stillage	36.5	Yen et al. (2012)
BCRC 22360	Batch	Lignocellulosic biomass hydrolysate	Ammonium sulfate, yeast extract	34.5	Yen and Chang (2014)
ATCC 204091	Batch	Nondetoxified liquid hydrolysate from pretreatment of wheat straw	Yeast extract	25.0	Yu et al. (2011)

## Mechanism of lipid biosynthesis

In yeast cells, lipids may be accumulate via two pathways: de novo (from acetyl-CoA and malonyl-CoA molecules) and ex novo (Beopoulos and Nicaud 2012). In the de novo method, saccharides or glycerol constitutes the substrates for lipids production (Papanikolaou and Aggelis 2011a), whereas in the ex novo biosynthesis, hydrophobic compounds serve as the substrates (Beopoulos et al. 2009). Culture media primarily containing glucose, sucrose, glycerol, and sugar waste raw materials such as molasses and different hydrolysates are used as sources of carbon for the production of lipids by the yeast *Rhodotorula glutinis* (Table 1).

In the de novo synthesis, the overproduction of intracellular lipids occurs after the depletion of nitrogen compounds from the culture environment, which is related to the activation of AMP deaminase. This enzyme catalyzes the decomposition of AMP to IMP and NH_4_^+^ ions, which constitute the additional source of nitrogen. A decrease in the adenosine monophosphate level disturbs the course of the Krebs cycle because this compound activates isocitrate dehydrogenase that catalyzes the transformation of isocitrate to α-ketoglutarate. Under such conditions, mitochondrion accumulates isocitrate that remains in balance with citrate, thanks to the activity of aconitase. After attaining a critical concentration, citric acid is transported from mitochondrion to the cytoplasm, where it is split by ATP-citrate lyase to acetyl-CoA and oxaloacetate. The first stage of fatty acid synthesis is carboxylation of acetyl-CoA, as a result of which malonyl CoA is produced. Then, a sequence of enzymatic reactions occurs, catalyzed by the complex of fatty acid synthase (FAS). The fatty acids produced can then be included in the pathway of triacylglycerol synthesis (Papanikolaou and Aggelis 2011a).

## Factors affecting on the biosynthesis of intracellular lipids

The biosynthesis of intracellular lipids by *Rhodotorula glutinis* is influenced by many factors. A significant role has the appropriate high C/N molar ratio in the culture medium. Saenge et al. (2011b) used glycerol as a carbon source for the production of lipids by *R. glutinis* TISTR 5159, and the C/N ratio in the medium remained in the range 35–85. The results obtained by the authors suggested that the optimal addition of glycerol for the production of the yeast biomass amounts to 8.5 % (C/N 60). When the glycerol content was increased to 9.5 % (C/N 85), highest lipid content was observed (approx. 42 %). Then, yeast was cultivated on the medium with an initial C/N ratio of 85 in a biofermenter tank. The highest lipid content (60.7 %) was obtained after 72 h, and it contained mainly oleic acid (45.75 %) and linoleic acid (17.92 %). The lipid yield after this time was 6.10 g/L.

The process of lipid biosynthesis by *R. glutinis* also depends on the type of carbon source found in the culture medium. Easterling et al. (2009) cultivated *R. glutinis* ATCC 204091 on media containing glucose, xylose, glycerol, glucose + xylose, glucose + glycerol, and xylose + glycerol. These compounds were added to the medium in such amounts that the initial C/N molar ratio amounted to 10. The intracellular lipids content varied, depending on the carbon source, and amounted from 10 (glucose and xylose) to 34 % (glucose + glycerol).

The acidity of the culture medium also has considerable effect on the lipids biosynthesis. Johnson et al. (1992) determined its influence during the cultivation of oleaginous strain of *R. glutinis* IIP-30 on medium containing 3 % of glucose as the source of carbon, 0.2 % of ammonium sulfate and 0.1 % of yeast extract as nitrogen sources. The highest lipid content (66 %) was obtained on the medium at pH 4.0, whereas at pH 3.0, 5.0, and 6.0, its content amounted to 12, 48, and 44 %, respectively.

The presence of dissolved oxygen in the culture medium constitutes another factor that determines the biosynthesis of intracellular lipids by yeasts. Yen and Zhang (2011a) observed that increasing the content of dissolved oxygen in the medium decreased the total amount of lipids produced in *R. glutinis* BCRC 22,360. When the oxygen level was established at 25 ± 10 %, the lipid content in the biomass was 62 %, whereas when the level was increased to 60 ± 10 %, it decreased to 52 %.

## Lipid biosynthesis from waste substrates

Studies showed that it is a possible to obtain microbial oils in media containing different waste products. Processes conducted on such substrates greatly increase the economic cost-effectiveness of SCO production and enable partial biodegradation of problematic industrial waste (Almazan et al. 1981; Alvarez et al. 1992; Cheirsilp et al. 2011, 2012; Gonzalez-Garcia et al. 2013; Lian et al. 2013; Liu et al. 2015; Louhasakul and Cheirsilp 2013; Saenge et al. 2011b; Schneider et al. 2013; Xue et al. 2008, 2010; Yu et al. 2011; Yen et al. 2012; Yen and Chang 2014).

Xue et al. (2008) used wastewater obtained from the production of monosodium glutamate as the nitrogen source. The medium was supplemented with glucose and *R. glutinis* was cultivated for 120 h. The obtained cellular biomass yield and lipid content amounted to 25 g_d.w._/L and 20 %, respectively. As a result of the process, a 45 % reduction of the chemical-oxygen demand indicator was obtained. Other authors (Gonzalez-Garcia et al. 2013) cultivated *R. glutinis* ATCC 204091 on medium prepared from distillery wastewater obtained from the production of tequila. After 144 h of the process, the lipid content amounted to 27 % and the COD index decreased by 84.44 %. Xue et al. (2010) used wastewater from starch production as culture medium. After 60 h of cultivation in 5-L biofermenter tank, the amount of cellular biomass obtained exceeded 60 g_d.w._/L and the lipids content amounted to 30 %. At the stage of the experiment, the culture was conducted in a 300-L biofermenter tank, using the same waste type as the culture medium, without prior sterilization and regulation of active acidity. The cellular biomass yield amounted to 40 g/L, and the participation of lipid remained at the level of 35 %, already after 30–40 h of the culture. After this time, an 80 % reduction in the chemical-oxygen demand indicator of the culture medium was noted. Yen et al. (2012) cultivated *R. glutinis* BCRC 22360 on medium containing crude glycerol obtained during biodiesel production and thin stillage collected from the brewing company. The process was carried out in a 5-L biofermenter tank, and the biomass yield and lipid content in the cellular biomass amounted to 14.8 g_d.w._/L and 36.5 %, respectively. Moreover, a study was conducted by Yen and Chang (2014) with the use of the same yeast strain on medium containing waste formed after the production of cellulose and hemicellulose (*lignocellulosic biomass hydrolyzate* (LCB)). Lipid content amounted to 34.3 % after cultivation on medium containing 6 % of reducing sugars.

Cheirsilp et al. (2011) cultivated a mixed culture of *R. glutinis* TISTR 5159 yeast and *Chlorella vulgaris* TISTR 8261 microalgae on media containing waste obtained from seafood processing plant and molasses obtained from sugar cane plant. It was determined that pure *R. glutinis* TISTR 5159 yeast culture exhibited a more rapid growth rate than the *Chlorella vulgaris* monoculture. However, in the case of a mixed culture, increased biomass yield and fat biosynthesis efficiency were observed compared to the separate culture of these microorganisms. This regularity could be the result of the syngergistic effect of these strains, consisting in the fact that the microalgae produced oxygen absorbed by yeasts, which biosynthesized lipids at the same time. The highest biomass yield (4.63 g_d.w._/L) and efficiency of microbiological oils (2.88 g/L) were obtained after 5 days of culture on medium with 1 % addition of molasses, under 5.0 klux light intensity.

## Fatty acid profile of lipids synthesized by *R. glutinis* and its uses

Lipids synthesized by *R. glutinis* contain primarily palmitic (C16:0), oleic (C18:1), linoleic (C18:2), and linolenic acids (C18:3). The main fatty acid included in the lipids synthesized by *R. glutinis* is oleic acid, and its percentage in the total pool of fatty acids may exceed above 60 %. The linoleic acid percentage ranges from above 5 to 25 %, and palmitic acid constitutes on average of above 10–30 %. Low percentage of the fat synthesized by these yeasts is characteristic of stearic acid (to 10 %); however, in certain strains, its content may reach up to 25 % (Table 2).

**Table 2:** Fatty acid profiles (%) of lipid synthesized by different strains of *Rhodotorula glutinis*

Strain	Fatty acid content																Carbon source	References
	C12:0	C14:0	C16:0	C16:1	C17:0	C18:0	C18:1	C18:2	C18:3	C20:0	C20:1	C22:0	C22:1	C22:6	C24:0	Sum: saturated/unsaturated		
AL_107_	1.0	1.1	21.4	1.4	1.7	4.9	58.6	3.6	2.2	2.2	–	1.6	0.3	–	–	33.9/66.1	Sucrose	Zlatanov et al. (2010)
ATCC 204091	–	0.4	24.3	0.2	0.2	10.1	53.2	6.8	1.1	0.3	–	0.7	–	–	2.4	39.1/60.6	Levoglucosan	Lian et al. (2013)
	–	0.5	18.1	1.2	0.1	24.4	44.6	5.6	1.6	1.6	–	0.6	–	–	1.7	47.0/53.0	Glucose	
IIP 30	–	–	23.4	–	–	3.5	34.7	25.1	11.4	–	–	–	–	–	–	26.9/71.2	Molasses	Johnson et al. (1995)
BCRC 22360	–	1.04	13.3	0.95	–	5.1	55.5	20.2	6.3	–	–	–	–	–	–	19.4/80.6	LCB	Yen and Chang (2014)
NCYC 154G	–	–	–	16.0	5.0	4.0	30.0	24.0	4.0	17.0	–	–	–	–	–	42.0/58.0	N-limitate medium with glucose, cultivation at 25 °C	Ratledge and Hall (1979)
ATCC 15125	–	–	13.9	–	–	5.75	46.54	26.57	3.37	–	–	–	–	–	–	19.65/76.48	Brewery effluents	Schneider et al. (2013)
TISTR 5159	0.18	1.04	20.37	0.83	1.38	10.33	47.88	7.31	0.85	–	–	1.5	–	–	1.03	35.83/56.87	Palm oil mill effluent	Saenge et al. (2011a)
CBS 20	–	–	26.9	–	–	10.1	37.9	22.9	1.9	–	–	–	–	–	–	37.0/62.7	Miscanthus	Mast et al. (2014)
CGMCC 2703	–	3.53	38.5	–	–	2.14	43.7	12.1	–	–	–	–	–	–	–	44.17/55.8	Undetoxified corncob hydrolysate	Liu et al. (2015)
*C. vulgaris* TISTR 8261 + *R. glutinis* TISTR 5159	0.98	2.98	40.52	1.15	1.1	17.15	21.3	1.41	–	–	–	1.44	8.89	–	3.08	67.25/32.75	Glycerol	Cheirsilp et al. (2012)

*C12:0* lauric acid, *C14:0* myristic acid, *C16:0* palmitic acid, *C16:1* palmitoleic acid, *C17:0* heptadecoic acid, *C18:0* stearic acid, *C18:1* oleic acid, *C18:2* linoleic acid, *C18:3* linolenic acid, *C20:0* arahinic acid, *C20:1* eicosanoic acid, *C22:0* behenic acid, *C22*:*1* erucic acid, *C22:6* docosahex-aenoic acid, *C24:0* lignoceric acid

Profile of fatty acids synthesized by the *R. glutinis* primarily depends on the yeast strain and composition of the culture medium (Zhang et al. 2011) (Table 2). However, the composition of the lipids can also be adjusted by modifying the molar ratio C/N in the culture medium (Braunwald et al. 2013), temperature of cultivation (Suutari et al. 1990), and by genetic modification of yeast (Shichang et al. 2013). A significant impact on the profile of fatty acids synthesized by the *R. glutinis* yeast has also time of cultivation (Mast et al. 2014; Zhang et al. 2011). Zhang et al. (2011) noted that increasing the time of cultivation *R. glutinis* ATCC 15,125 yeast increased content of unsaturated fatty acids, from 46 (0 h) to 63.1 % (233 h). At this time, oleic and linoleic acids content increased from 26.9 and 8.5 to 43.8 and 12.7 %, respectively.

Significant impact on the profile of fatty acids has also the molar ratio of carbon to nitrogen (C/N) in the culture medium. Braunwald et al. (2013) observed that the content of saturated fatty acids C16:0 and C18:0 in the biomass of *R. glutinis* ATCC 15125 was the lowest after cultivation in the medium with the initial C/N 20. Also, in this case, authors observed the highest concentration of oleic C18:1 (39.9–44.4 %) and linoleic acids C18:2 (31.2–42.3 %). In contrast, the highest content of linolenic acid (6.54–7.35 %) was determined in yeast cells cultivated in media with a high C/N ratio equal 70 and 120, while in the medium with the initial C/N 20 was significantly lower (3.67–3.97 %).

Composition of fatty acids synthesized by *R. glutinis* is also dependent on temperature of cultivation. Changes in the proportions of fatty acids are one of the factors of yeast adaptation to life in environments with different temperatures. At a lower temperature, yeasts synthesize more unsaturated fatty acids, which is associated with changes of the cell membranes (Zlatanov et al. 2010). Suuta et al. (1990) found that the *Rhodosporidium toruloides* VTT-C-132 82 (teleomorph stages of *R. glutinis*) synthesized the largest amount of linoleic acid (approx. 22 %) at 10 °C. The content of this acid after cultivation at 40 °C was only approx. 10 %. Lipids synthesized by *R. glutinis* can be also enriched in linoleic acid through genetic modification. Shichang et al. (2013) used for this purpose implantation of nitrogen ions. The obtained mutant D30 synthesized almost 3-fold more linoleic acid (27 %) compared to the parental strain *R. glutinis* 31,596 (9.93 %), while significantly reduced oleic acid (from 61.8 to 49.3 %) and palmitic acid (from 5.66 to 11.0 %).

Due to the participation of individual fatty acids in the lipids synthesized by *R. glutinis*, researchers have indicated the possibility of using these yeasts as a source of substrates for biodiesel production (Dai et al. 2007; Liu et al. 2015; Mast et al. 2014; Saenge et al. 2011a; Schneider et al. 2013; Xue et al. 2010; Yen and Zhang 2011b; Zhang et al. 2011). Biodiesel is defined as a fuel consisting of fatty acid monoalkyl long-chain esters, most commonly methyl esters. The ecological aspect of the fuel explains the considerable growth in the interest its usage. Biodiesel is fully renewable and biodegradable. Its usage favorably influences the state of the environment, primarily because of its decreased emission of greenhouse gasses to the atmosphere (Adamczak et al. 2009). Depending on the type of raw material used for its production, biofuel is classified as three types and are as follows: first-generation biodiesel produced from plant oils such as rapeseed and soybean oil, second-generation biodiesel produced from oily nonfood raw materials (e.g., *Jatropha*), and third-generation biodiesel produced from the lipids of microbiological origin (Schneider et al. 2013). Currently, third-generation biodiesel is not produced at industrial level because their microbiological synthesis is expensive (Zhang et al. 2014). Therefore, further study should concentrate on lowering its production costs. This aim can be achieved by genetically modifying yeasts in order to increase their biosynthetic efficiency or by using waste products as components of the culture media (Kot et al. 2015).

## Carotenoid biosynthesis by *Rhodotorula glutinis*

Carotenoids belong to the group of natural pigments found in fruits, vegetables, fish, eggs, and oil (Rao and Rao 2007). Additionally, they are synthesized by certain microbes, including *R. glutinis* yeast (Table 3) (Perrier et al. 1995). They are characterized by yellow, orange, or red coloration. Until now, approximately 750 compounds of this type have been identified (Maoka 2011), out of which 50 compounds exhibit provitamin A activity (Fraser and Bramley 2004). Humans are unable to biosynthesize carotenoids, and therefore, they must be supplied with diet (Woodside et al. 2015). These compounds are highly soluble in fats but they do not dissolve in water. Carotenoids exhibit health promoting activity toward human body. Thanks to their antioxidant properties, they protect the skin against the UV light. They possess antioxidative effect against free radicals as well as reactive oxygen species. They strengthen the immune system and accelerate wound healing. Some carotenoids may be protective in eye disease because they constitute vitamin A precursors (Krinsky and Johnson 2005; Rao and Rao 2007).

**Table 3:** Efficiency of carotenoid biosynthesis by different strains of *Rhodotorula glutinis*

Strain	Cultivation method	Carbon source	Nitrogen source	Carotenoid biosynthesis efficiency (mg/L)	References
NCIM 3353	Batch	Molasses	Malt extract	24.1	Bhosale and Gadre (2001a)
			Yeast extract	42.6	
			Ammonium sulfate	14.4	
DBVPG 3853	Batch	Concentrated rectified grape must		5.95	Buzzini and Martini (1999)
DBVPG 3853	Batch	Concentrated rectified grape must	Yeast extract	6.97^a^	Buzzini (2000)
C2.5t1	Batch	Glycerol	Yeast extract	14.92	Cutzu et al. (2013)
CCY 20-2-26	Fed-batch	Glucose	Ammonium sulfate	23.34	Marova et al. (2010)
MT-5	Batch	Detoxified loquat kernel extract	Peptone	72.36	Taskin and Erdal (2010)
MT-5	Batch	Glucose	Waste chicken feathers, yeast extract	92	Taskin et al. (2011)
TISTR	Batch	Sweet potato extract	Dried mung bean flour	3.48	Tinoi et al. (2005)

^a^Only β-carotene content provided

Carotenoids are used in various industrial sectors as components of cosmetics (Anunciato and da Rocha Filho 2012) and additives to fodders for livestock (Chatzifotis et al. 2005) and fish (Gouveia et al. 2003). They are also commonly used in food industry as food pigments (Carocho et al. 2015). According to the data published in the report “The Global Market for Carotenoids” in 2014, the world’s carotenoid market has achieved a value of 1.5 bn USD, and it is forecast that in 2018, it will increase to 1.8 bn USD (BCC Research 2016). The increasing consumer awareness on the negative effect of synthetic pigments on human health and on the healthy diet trend causes increasing interest in natural pigments (Panesar 2014). The use of microorganisms as bioreactors for the production of carotenoids can constitute an alternative for chemical synthesis (Del Campo et al. 2007). Microbiological synthesis is a more effective method in comparison to extraction from vegetables or chemical synthesis. The most important advantages of the process include the possibility to decrease its costs by the use of improved strains and inexpensive (often waste) carbon and nitrogen sources in culture media (Buzzini 2000).

## Mechanism of carotenoid biosynthesis

**Fig. 1:**
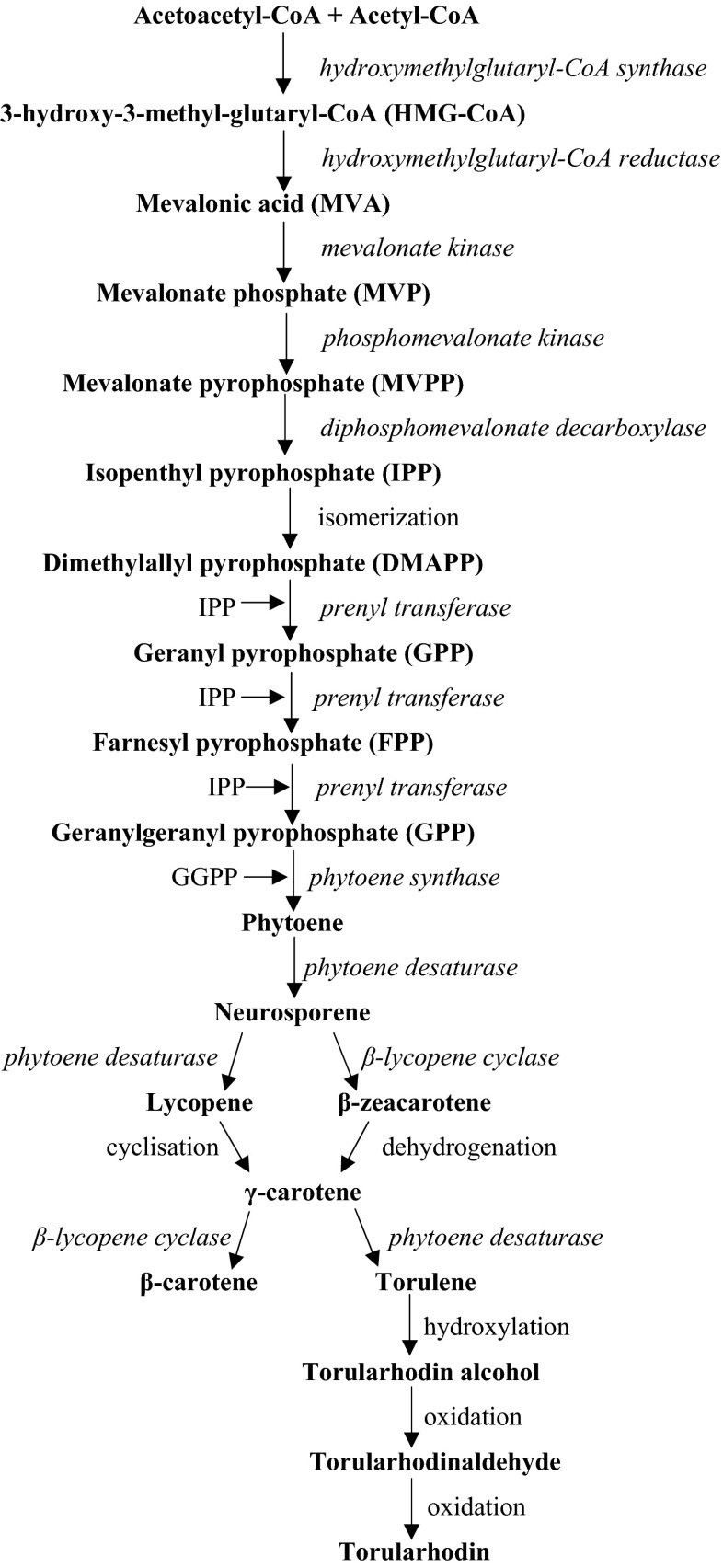
Carotenoid biosynthetic pathway in *Rhodotorula* species (elaborated on the basis of Frengova and Beshkova 2009; Goodwin 1980; Hayman et al. 1974; Johnson and Lewis 1979; Simpson et al. 1964; Squina and Mercadante 2005)

*Rhodotorula glutinis* are capable of synthesizing β-carotene, torulene, and torularhodin, the percentage of which depends on the cultivation conditions (Latha et al. 2005). The first stage of carotenoid biosynthesis includes the conversion of acetyl-CoA to 3-hydroxy-3-methylglutaryl-CoA with the participation of hydroxymethylglutaryl-CoA synthase. Subsequently, HMG-CoA is transformed to mevalonic acid (MVA) by specific reductase. As a result of subsequent changes, the compound is subjected to phosphorylation in a reaction catalyzed by specific kinases and decarboxylation to isopentenyl diphosphate (IPP). The IPP isomerization reaction leads to the formation of dimethylallyl pyrophosphate (DMAPP) and then to the DMAPP as a result of the addition reaction of three IPP molecules. These reactions lead to the formation of geranylgeranyl pyrophosphate (GGPP) containing 20 carbon atoms. Condensation of two GGPP molecules is catalyzed by phytoene synthase, leading to the formation of phytoene (first 40-carbon product of the pathway). This compound is then converted to neurosporene with the participation of phytoene desaturase. Neurosporene molecule may be transformed to lycopene or β-zeacarotene (Goodwin 1980; Hayman et al. 1974; Simpson et al. 1964). A second reaction probably takes place due to the presence of inhibitors, such as diphenylamine or in the case of environmental stress (Johnson and Lewis 1979). Then γ-carotene is formed as a result of lycopene cyclization. This compound can be produced in yeast cells also as a result of β-zeacarotene dehydrogenation reaction (Hayman et al. 1974). The γ-carotene cyclization reaction, catalyzed by β-lycopene cyclase, leads to the formation of a β-carotene molecule. Moreover, the γ-carotene molecule constitutes a precursor of torulene synthesis. Torularhodin is produced as a result of further transformations of torulene, consisting in hydroxylation and oxygenation reactions (Goodwin 1980) (Fig. 1).

## Influence of selected factors on the efficiency of biosynthesis and total carotenoids profile

The efficiency of carotenoid biosynthesis by *R. glutinis* depends on numerous factors. The types of carbon and nitrogen sources have significant influence, and their preferred form and concentration may differ depending on the yeast strains (Buzzini and Martini 1999; El-Banna et al. 2012; Latha et al. 2005; Panesar et al. 2013). Due to this, selection of the correct carbon and nitrogen source constitutes one of the most important tasks for the determination of the culture medium composition.

One of the factors that stimulate the biosynthesis of carotenoid pigments is light. An increase in the content of these compounds under illumination conditions probably stems from the higher activity of enzymes involved in the biosynthetic pathway (Frengova and Beshkova 2009). An example here is the study of Zhang et al. (2014), in which *R. glutinis* cultures were subjected to illumination with LED lamps (800–2400 mol/m^2^ s). The carotenoid productivity was observed to be maximum (2.6 mg/L) when the culture was illuminated with three LED lamps, which constituted over 2-fold increase compared to the control culture (1.2 mg/L). In this case, inhibitory influence of illumination on the yeast’s growth was not observed. Cellular biomass yields after 60 h for the control culture and the culture illuminated with two and three LED lamps amounted to 15.9, 17.4, and 17.7 g/L, respectively. Bhosale and Gadre (2002) carried out *R. glutinis* NCIM 3353 no. 32 yeast cultures at a constant illumination with an intensity of 1000 lx. This amount of illumination used considerably slowed down the growth of the studied yeast strain. The carotenoid concentration decreased from 125 to 83 mg/L, after 72 h of cultivation in 30 °C. Cultures were also conducted beginning illumination at the late logarithmic growth phase. This measure increased the carotenoid productivity to 198 mg/L. Sakaki et al. (2001) conducted a study on the influence of white light (3500 lx) on the carotenoid biosynthesis using a wild strain of *R. glutinis* no. 21. It was determined that the culture illumination intensified the production of all carotenoid fractions, in particular torularhodin. The β-carotene, torulene, and torularhodin production increased from 3.6, 29.2, and 7.9 to 4.2, 32.2, and 14.2 mg/100 g_d.w._, respectively.

Temperature is an important parameter that regulates the biosynthesis of carotenoids, and the activity of the enzymes that participate in their production process depends upon it (Frengova et al. 1995). β-Carotene synthesis by *R. glutinis* is increased at lower temperature. This situation is reversed in the case of torulene and torularhodin; their production is increased at higher temperatures (Nakayama et al. 1954). Perhaps, at lower temperature, the enzymes engaged in the torulene biosynthesis are less active (Bhosale 2004). The study of Simpson et al. (1964) demonstrated that after 21 days of culture at a temperature of 5 °C, the β-carotene content in the biomass of *R. glutinis* amounted to 64 % and torulene and torularhodin to 4.4 and 4.8 %, respectively. After 12 days of culture of the same strain at 25 °C, these values amounted to 25.2, 27.8, and 24.3 %, respectively. Similar results were obtained by Frengova et al. (1995). When a coculture of the yeast *R. glutinis* 22P and the bacteria *Lactobacillus helveticus* 124 was cultivated at 20 °C, β-carotene, torulene, and torularhodin content amounted to 19.0, 22.8, and 56.0 %, respectively. At 35 °C, considerable increase in the production of torularhodin was observed (78.3 %), whereas that of β-carotene and torulene was low and amounted to 9.6 and 9.0 %. Moreover, an elevation of the culture temperature from 20 to 35 °C led to a significant increase in the productivity of carotenoid biosynthesis—its content increased from 145.4 to 280.0 μg/g_d.w._

*R. glutinis* is an aerobic microorganism, and thus ensuring correct aeration is the necessary condition for carotenoid biosynthesis (Saenge et al. 2011b). Frengova and Beshkova (2009) have stated that in the case of yeasts of the genus *Rhodotorula*, the rate at which the culture is mixed should be in the range from 180 to 190 rpm, and the air flow should range from 0.5 to 1.9 L/min. Aksu and Eren (2007) have reported that increase in the aeration rate from 0 to 2.4 vvm significantly increased carotenoid biosynthesis by *R. glutinis*. The total biosynthetic efficiency increased from 63.4 to 105.8 mg/L after 10 days of cultivation.

Supplementing the culture medium with certain metal ions can increase carotenoid biosynthesis by *R. glutinis* (Bhosale and Gadre 2001b; El-Banna et al. 2012). El-Banna et al. (2012) studied the influence of magnesium, zinc, iron (II), copper, manganese, and calcium salts on the efficiency of the process. The highest content of these compounds (638 μg/g_d.w._) in the biomass was reported when the culture medium was supplemented with 0.1 % of zinc sulfate (VI). In comparison to the control medium (292 μg/g_d.w._), almost 2-fold increase in carotenoid content was reported in the yeast biomass. Also, enriching the culture medium with iron (II) sulfate (VI) and copper sulfate (VI) increased its content to 460 and 674 μg/g_d.w._, respectively.

The presence of different solvents and chemical compounds in the culture medium can also intensify the carotenoid production by *R. glutinis*. Saenge et al. (2011b) studied the influence of the addition of three surfactants, such as Tween 20, Tween 80, and gum arabic, on the carotenoid biosynthesis by the yeast *R. glutinis* TISTR 5159. It was determined that the presence of Tween 20 in the culture medium had the most efficient influence in increasing carotenoid productivity (108.94 mg/L) compared to the control culture (65.86 mg/L). The presence of chemical compounds has also impact on proportion of the synthesized carotenoids. It was reported that the addition of ethanol to the culture medium stimulates production of β-carotene and torulene but inhibits the synthesis of torularhodin (Bhosale 2004). Kim et al. (2004) reported that the addition of phenol at a concentration of 500 ppm to the medium increased the production of β-carotene by 35 %. This measure limited the torularhodin synthesis, whereas the torulene content remained at a stable level.

The efficiency of biosynthesis and composition of the fractions of carotenoids synthesized by the *R. glutinis* yeast mainly depends on the strain and the culture conditions. Nowadays, great potential in this area provides also the techniques of genetic modification. Bhosale and Gadre (2001a) used UV radiation (250–280 nm) to modify wild strain of *R. glutinis* NCIM 3353. Selected for further study, yellow-colored mutant no. 32 produced 120-fold more β-carotene than the parent strain. β-Carotene was 82 % (*w*/*w*) of the total carotenoid content, whereas parent strain produced only 14 % of this compound. Sakaki et al. (2000) conducted a mutagenizing of *R. glutinis* strain no. 21 isolated from soil by UV radiation (254 nm). As a result of this procedure, authors obtained the mutant TL/21. This yeast synthesized 3-fold more torularhodin (4.3 mg/100 g_d.w._) than the parent strain (1.5 mg/100 g_d.w._).

## Industrial use of carotenoids produced by *R. glutinis*

β-Carotene is the most desired carotenoid type, commonly used as pigment in foods and diet supplements (Carocho et al. 2015; Schierle et al. 2004). Currently, torulene and torularhodin are not commercially used. It is generally known that torulene (C_40_H_54_) exhibits properties of provitamin A and antioxidative effect (Maldonade et al. 2008). It was determined in an in vitro study that torularhodin (C_40_H_52_O_2_), carboxylated torulene derivative, has greater capacity to neutralize free radicals compared to β-carotene (Sakaki et al. 2001). Scarce scientific publications have indicated the possibility to use torulene and torularhodin as components of cosmetics and food (Zoz et al. 2015), and as ingredients of drugs (Ungureanu and Ferdes 2012). Toxicity studies conducted on rats demonstrated that β-carotene, torulene, and torularhodin produced by *R. glutinis* DFR-PDY yeasts can be used as safe food additives (Latha and Jeevaratanm 2012). The capacity of *R. glutinis* to synthesize carotenoids can be also used for medical purposes, for example, dried and powdered *R. glutinis* NCIM 3353 yeasts biomass added to the fodder for rats. It was determined that it exhibited protective effects against the precancerous lesions of the liver induced by *N*-nitrosodimethylamine (Bhosale et al. 2002). Moreover, torulene and torularhodin inhibit the growth of prostate cancer (Du et al. 2016). Torularhodin can be also used as a neuroprotective agent against H_2_O_2_-induced oxidative stress, due to its strong antioxidant activity (Wu et al. 2015).

## Biosynthesis of phenylalanine ammonia lyase by *R. glutinis*

*R. glutinis*, depending on the culture conditions, has the capacity to synthesize different types of enzymes that can be used in various industrial sectors. It was determined that the biomass of these yeasts can be source of lipases (Hatzinikolaou et al. 1999; Khayati and Alizadeh 2013; Papaparaskevas et al. 1992), α-l-arabinofuranosidase (EC 3.2.1.55) (Martínez et al. 2006), invertase (EC 3.2.1.26) (Canli et al. 2011; Rubio et al. 2002), pectinases, and tannin acyl hydrolase (EC 3.1.1.20) (Taskin 2013). However, researches have focused primarily on the possibility to obtain phenylalanine ammonia lyase (E.C.4.3.1.5). As a result of the effect of this enzyme, it is possible to obtain l-phenylalanine, which constitutes the substrate for aspartame production (D’Cunha et al. 1996a, 1996b; Zhu et al. 2014).

Phenylalanine ammonia lyase (PAL) catalyzes the nonoxidative process of phenylalanine transformation to trans-cinnamic acid and ammonia (D’Cunha et al. 1996a, 1996b). Under controlled conditions, this reaction may also take place in a reverse direction (Takac et al. 1995). In the food industry, this enzyme is used in the production of l-phenylalanine and para-hydroxycinnamic acid (Cui et al. 2015), and in medicine in phenylketonuria therapy (Longo et al. 2014; Sarkissian and Gámez 2005) and neoplastic cancers in mice (D’Cunha 2005). Furthermore, the activity of PAL is used to determine the concentration of l-phenylalanine in blood plasma (Watanabe et al. 1992).

The enzyme phenylalanine ammonia lyase occurs in common in microorganism cells. It was isolated from the cells of *Streptomyces verticillatus* (Bezanson et al. 1970), *Rhizoctonia solani* (Kalghatgi and Subba Rao 1975), and *Neurospora crassa* (Sikora and Marzluf 1982). However, the largest producers of PAL are yeasts of the genus *Rhodotorula*. In the yeast cells, this enzyme participates in the absorption of phenylalanine as the source of carbon and nitrogen (Gientka et al. 2006).

Numerous research teams (D’Cunha et al. 1996b; Takac et al. 1995; Yamada et al. 1981) conducted studies on the obtainment of l-phenylalanine in the presence of PAL originating from the cells of *R. glutinis*. To grow yeast cells, media containing easily assimilated nutrient sources are used. Glucose is used as the source of carbon and yeast extract as that of nitrogen. Culture media are also supplemented with zinc, magnesium, iron, cobalt, and calcium salts. The grown yeast cells are then transferred to a medium, in which phenylalanine ammonia lyase induction occurs. The following compounds act as inducers: l-phenylalanine, d,l-phenylalanine, l-tyrosine, d,l-tyrosine, and l-isoleucin. Typical l-phenylalanine dose, which induces PAL production, is 0.4–0.5 %; higher concentrations do not have a significant effect on activity of the enzyme. Yeast cultures on inductive medium is conducted to the moment, when the phenylalanine ammonia lyase activity achieves the level of at least 0.2–2.0 U/ml (Gientka et al. 2006).

Bioconversion of trans-cinnamic acid to L-phenylalanine is carried out with isolated enzyme or directly with yeast cells rich in PAL (Gientka et al. 2006). One of the factors that determine the course of the process is the acidity of the environment. Yamada et al. (1981) and Evans et al. (1987) have stated that the optimal pH for the bioconversion process of cells of *R. glutinis* is 10.0, whereas El-Batal et al. (2000) concluded that for *R. glutinis* mutants, this value is equal to 11.0. The pH value of the reaction environment should not be less than 9.0 because the deamination of l-phenylalanine to trans-cinnamic acid takes place under this value (Gientka et al. 2006).

In order to obtain the maximum efficiency of the bioconversion process, it is important to determine the optimal concentration of trans-cinnamic acid in the reaction environment. The reaction occurred under the conditions of its excess; however, it was determined that very high concentration of trans-cinnamic acid inhibits the activity of phenylalanine ammonia lyase. The terminal concentration of the acid in the reaction environment should not exceed 50 mM (Takac et al. 1995).

The factor determining to a large extent the activity of phenylalanine ammonia lyase is the temperature. Takac et al. (1995) determined that the bioconversion process of trans-cinnamic acid to l-phenylalanine takes place most efficiently at a temperature of 30 °C. On the other hand, increasing the temperature to 40 °C decreased the concentration of the amino acid by approximately 50 %. The bioconversion process is further influenced by the presence of different types of chemical compounds in the reaction medium. The same research team noticed that the addition of sodium glutamate and penicillin increased the activity and stability of phenylalanine ammonia lyase during the bioconversion process. On the other hand, the presence of Cl^−^ ions in the culture environment has inhibitory effect on the process.

The elaboration of an efficient method that warrants the maintenance of PAL stability and activity, so that the enzyme could be used in a constant process, constitutes an important issue. D’Cunha et al. (1996b) observed that immobilization of *R. glutinis* NCYC 61 cells did not prevent the degradation of phenylalanine ammonia lyase, which made it impossible to use the yeast cells again. However, it was noticed that the addition of Mg^2+^ ions and glycerol to the reaction environment stabilized PAL. The addition of 4 mM of MgSO_4_ and 10 % glycerol enabled to obtain l-phenylalanine in nine production cycles, whereas the immobilized enzyme lost its activity in the fourth production cycle. D’Cunha (2005) devoted the next study to obtain increased level of phenylalanine ammonia lyase from the culture of *R. glutinis*. The process consisting in the use of entire yeast cells had low efficiency due to the low permeability of the cellular membrane for l-phenylalanine, the effect of ultrasonication, detergents, and enzymes on the increase of PAL activity were tested. Ultrasonication turned out to be the most efficient method, with which the enzyme activity could be increased 10 times compared to the control.

## Conclusions and future prospects

Recently, the use of products synthesized microbiologically has been increased in various industrial sectors. Due to its capacity to produce metabolites, *Rhodotorula glutinis* may become an important link of development in modern biotechnology. This yeast belongs to the group of oleaginous microorganisms and is capable to producing and accumulating even 60 % of lipids in dry cellular substance (Dai et al. 2007). Due to the participation of individual fatty acids, these lipids can be used as substrates for third-generation biodiesel production. Dynamically increasing production of unconventional fuels such as biodiesel is the result of decrease of nonrenewable sources such as petroleum and environmental care. The use of *R. glutinis* yeast as bioreactors for the production of microbial oils is currently limited by expensive production. It may be assumed that further study will focus on increasing the biosynthetic efficiency by optimization cultivation conditions and by genetically modifying the organisms. Moreover, another direction of research should concentrate on the use of extracted biomass, which after removal of solvents could be added to animal feed. This use of waste yeast biomass, which contains mainly proteins and polysaccharides, additionally increases profitability of the microbial production of SCO.

In recent years, there has been an increased consumer knowledge of negative impact of synthetic colorants on health. Therefore, researchers are looking for new producers of natural dyes (Torres et al. 2016). *R. glutinis* are capable of synthesizing β-carotene, and two other carotenoids—torularhodin and torulene. These compounds have not been detected in foods, and probably of this, their effects on the human health have not been investigated and described yet. However, taking into account their chemical structure and properties, it seems clear that these two substances can be used as food additives (Zoz et al. 2015). Furthermore, torulene and torularhodin have the potential to be used in medicine and pharmacy. The first direction of their use may be the prevention of prostate cancer (Du et al. 2016). Torularhodin has strong antimicrobial properties, and it may become a new natural antibiotic (Keceli et al. 2013; Ungureanu and Ferdes 2012). Antimicrobial properties of torularhodin can be also used in the production of films for coating of medical implants (Ungureanu et al. 2014, 2016). These examples describe the prospects for the use of carotenoids synthesized by the *R. glutinis*; however, it is necessary to perform additional nutritional and toxicological tests that will allow for the introduction of torulene and torularhodin on the commercial market.

*R. glutinis* yeast can be source of various types of enzymes that can be used in various industrial sectors, especially phenylalanine ammonia lyase. This enzyme is accumulated intracellularly, and thus, the most promising appears to be the use of whole cells in the biotransformation of trans-cinnamic acid to L-phenylalanine. This would reduce the costs associated with the disintegration and enzyme secretion, after biotransformation of yeast biomass can be used as an animal feed additive.

This bibliographical review has shown that *R. glutinis* yeasts have great potential for industrial applications. Cultivation of these yeasts is independent of the climate and season, and the production cycle is short. In addition, the *R. glutinis* yeasts are capable to metabolize different substances as sources of carbon and nitrogen, so the use of many waste materials as components of culture media is possible. As a result, the biodegradable industrial waste with simultaneous production of yeast biomass containing valuable nutrients is possible (Kieliszek et al. 2015). However, still it is necessary to conduct studies on reducing the cost of obtaining lipids, carotenoids, and enzymes from *R. glutinis* yeast biomass for the industrialization these processes.
